# Multi-faceted nutritional interventions are imperative to reduction of stunting among children in low- and middle-income countries

**DOI:** 10.3389/fnut.2025.1479850

**Published:** 2025-09-09

**Authors:** Amy R. Sharn, Elena Oliveros, Stephanie Lai, Claudia P. Sanchez, Mary Jean Villa-Real Guno, Clara Rojas Montenegro

**Affiliations:** ^1^Global Medical Affairs and Research, Abbott Nutrition, Columbus, OH, United States; ^2^Nutrition Science, Abbott Nutrition, Granada, Spain; ^3^Global Medical Affairs and Research, Abbott Nutrition, Bogotá, Colombia; ^4^Ateneo de Manila University School of Medicine and Public Health, Pasig, Philippines; ^5^Universidad del Rosario-Escuela de Medicina, Bogotá, Colombia

**Keywords:** stunting, pediatric malnutrition, program evaluation, child health, nutrition programs

## Abstract

Worldwide, 1 in 5 children under 5 years experiences undernutrition; most commonly in in low- and middle-income countries. Inadequate nutrient and energy intake places children at risk of stunted growth, which is associated with delayed development, increased mortality, and reduced productivity in adulthood. We investigated global approaches for managing stunting in young children (ages 1–5 years) by reviewing research on nutrition-focused interventions and public health frameworks. Our aim was to identify components of effective nutritional care and monitoring. We screened 1,636 studies, reviewed 207 abstracts and full-texts, and included 9 studies for final analysis. These studies, conducted in China, Colombia, Guatemala, Haiti, India, Mexico (*n* = 2), Peru, and Vietnam evaluated clinical outcomes such as anthropometrics and dietary intake. Most interventions included caregiver nutrition education (*n* = 7), but none used routine and frequent nutrition screening; only 4 included frequent follow-ups, 3 assessed breastfeeding frequency, and 4 used macronutrient and micronutrient supplementation when indicated. Economic outcomes were reported in 4 studies, while process and clinical outcomes were commonly reported (*n* = 7). Based on our review, effecting stunting interventions should include: (i) routine screening of every child for nutritional risk based on WHO and UNICEF guidance, (ii) caregiver-targeted nutrition education (iii) supplementation with macro- and micronutrients as needed, and (iv) regular follow-up to monitor growth and nutritional status. Although the evidence base was small, stringent inclusion criteria focused on community-based, multi-component interventions. This highlights the need for expanded implementation research, particularly in under resourced regions. Comprehensive, multi-level strategies are essential to address the long-term health risks of pediatric undernutrition.

## 1 Introduction

Pediatric undernutrition remains a significant challenge today, particularly in low- and middle-income countries. More than one in five children under the age of five are affected by undernutrition, primarily due to inadequate intake of energy, protein and key micronutrients essential for healthy growth (1, 2). Beyond nutrient intake, early growth is also influenced by infections, subclinical pathogen exposure, and alterations in gut microbial communities during the first 1,000 days of life. Additionally, infections, subclinical pathogen carriage, and the metabolic impact of ‘dysbiotic’ commensal gut microbial communities can also influence undernutrition during the first 1,000 days (3). Infant gut microbiota affects the somatotropic axis through regulation of Insulin-like Growth Factor-1 (IGF-1) and growth hormone production, thereby affecting growth (3). These biological and environmental contributors disrupt hormonal pathways such as the somatotropic axis, further exacerbating growth faltering.

Stunting, defined as impaired linear growth or failure to achieve expected height for age, affected 22.3% (148.1 million) children under five globally in 2022 (4). Although some global regions have seen progress, stunting remains highly prevalent in low- and middle-income countries, with an estimated 95% of affected children residing in Asia or Africa. Latin America and the Caribbean also continue to face high rates, with Central America reporting prevalence as high as 16.9% (4). This persistence reflects not only nutritional deficits but also broader socio-economic inequalities and gaps in public health infrastructure (5, 6).

Despite widespread recognition of the 1,000 day window as the most critical period for stunting prevention (5, 7–9), growth faltering can also occur in later childhood (10, 11). Evidence suggests that nutritional interventions beyond infancy may still improve both linear growth and cognitive development (11–15), though catch-up growth after age two must be approached cautiously due to its association with increased risk for overweight, obesity, and cardiometabolic disease in adulthood (16, 17). Therefore, stunting prevention strategies must ensure nutrient adequacy without promoting excessive energy intake. Rapid catch-up growth after age two has been particularly linked with these long-term risks, underscoring the need for nutrient and energy adequacy (16, 17).

Undernutrition in early life has both immediate and long-term consequences, including impaired immune function, increased infection-related mortality (13), and delayed physical and cognitive development which may impact adulthood performance, resulting in shorter adult stature, reduced educational attainment, increased risk of chronic diseases, and lower economic productivity (2, 5, 16). This low individual productivity is a major barrier to individual and national development (16, 18, 19).

Global health frameworks such as the WHO and UNICEF conceptual models highlight the need for multi-level, multi-component strategies to prevent and treat undernutrition and stunting (19–21). These frameworks emphasize the importance of maternal nutrition, early initiation of breastfeeding, timely complementary feeding, and community-level improvements in hygiene and healthcare access (Table 1). Globally, only 44% of infants aged 0–6 months are exclusively breastfed, and fewer than one in four children aged 6–23 months meet the minimum criteria for dietary diversity and feeding frequency appropriate for their age (8). UNICEF guidance emphasizes the importance and efficacy of caregiver education, vitamin and mineral supplementation in nutrient-poor settings, and access to fortified foods aligned with national standards (9). They also call for implementation of targeted, context-specific programs that integrate caregiver education, access to fortified foods or supplements, and regular growth monitoring.

**Table 1:** Childhood stunting: socioecological levels for treatment opportunities.

Socioecological level	Treatment target/leader
Individual	Child with stunting
Interpersonal	Caregiver
	Community Health Worker
	Healthcare Professional
Organizational	NGO Programming + Staff
	NGO Leadership
Community	NGO Sites
	Neighborhood Environment
Structures + systems	Ministry of Health
	Economy

NGO, Non-Government Organization.

Despite significant policy momentum, including the 2012 World Health Assembly resolution to reduce stunting by 40% by 2025 and the UN Sustainable Development Goal to end all forms of malnutrition by 2030, progress has been insufficient (4, 18, 20). A 2021 Lancet series reinforced the need for scalable, locally adapted programs that address nutritional needs at multiple socio-ecological levels, from household feeding practices to national health policies (18, 22, 23).

Community-based nutrition interventions offer a promising delivery model, particularly for reaching under-resourced populations. These programs can incorporate low-cost supplements, caregiver counseling, child health monitoring, and behavior change strategies that engage families and local networks (24–27). When designed with cultural and contextual sensitivity, such interventions may also yield economic benefits, with some estimates suggesting returns on investment as high as 17% and benefit–cost ratios of 5:1 (28).

Despite these promising models, large-scale implementation remains challenging. The *Lancet* maternal and child undernutrition series (2021) highlighted uneven progress toward stunting reduction across low- and middle-income countries and emphasized the need for multi-sectoral efforts aligned with the UN Sustainable Development Goals (18). Earlier work from the 2013 *Lancet* series had already underscored the potential for greater private sector involvement in shaping affordable, scalable nutrition interventions (18). However, limited research exists on how to operationalize such partnerships effectively. The 2021 series further emphasized the need for robust implementation science and adaptive strategies that respond to what works in specific local contexts, underscoring persistent gaps in translating global frameworks into sustainable programs (29).

### 1.1 Aim for literature review

Although several studies have evaluated stunting prevention and intervention strategies, there remains limited research on the effectiveness and implementation of comprehensive, community-based nutritional care programs for young children. This review aims to address this gap by synthesizing evidence from studies that implemented multi-component community nutrition interventions. We specifically sought to understand which strategies have demonstrated clinical, health, or economic benefit, and how these strategies were integrated and sustained in community settings. The review was guided by the WHO and UNICEF frameworks and focused on practical implementation of nutritional care at multiple levels, from caregivers to health systems, to support improved growth outcomes among children at risk for or with stunting.

## 2 Methods for literature review

For our literature review, we searched publications from January 1990 to September 2023 from databases Allied & Complementary Medicine™, Embase®, EMCare®, FSTA®, and MEDLINE®. MeSH terms included “nutritional interventions,” “nutritional supplement,” “pediatric,” “malnutrition,” and “stunting.” Criteria for study inclusion were children aged 0–10; community-based; use of a nutrition supplement; nutrition education; clinical, health, or economic outcomes. Additional studies authors were aware of that were not already identified by the search were added manually (manual search) if they met study criteria. Although stunting most commonly originates in the first 1,000 days of life, this review included studies with participants aged 0–10 years to capture long-term outcomes of early-life interventions and evaluate follow-up impacts in later childhood. Notably, most included studies enrolled children younger than 5 years, aligning with the early-life focus of stunting prevention. Despite targeted manual searching, no studies from Sub-Saharan Africa met our predefined inclusion criteria, which required both nutrition education and supplementation components with stunting-specific outcomes. As such, all included studies were from Latin America and Asia. Studies were compared by their intervention components among 5 categories:

- *Nutrient Intake Intervention*: nutrition provided as part of the intervention.
- *Education*: topics included as part of the intervention.
- *Education Mode*: active (synchronous) or passive (asynchronous education such as flyers or posters).
- *Other Resources Provided*: other resources such as deworming program, farm animals, farming equipment, community gardens, etc.
- *Follow-Up*: non-frequent (greater than monthly/more than 4 weeks); frequent (at least once per month).

Trends were identified if the majority within an intervention component category had similar findings. While several included studies reported additional outcomes such as wasting and underweight which are expected, common concurrent conditions when evaluating nutritional recovery conditions, our review was limited to evaluating effects on stunting (height-for-age z-scores). This paper presents a narrative review based on a systematic search to identify representative interventions focused on stunting outcomes, rather than an exhaustive synthesis. The use of strict inclusion criteria, specifically requiring studies to combine nutrition education with supplementation and report stunting-related outcomes, was intentional to ensure focus, but may have limited the number of studies eligible for inclusion.

## 3 Results of our focused literature review

We initially identified 1,636 studies, narrowed the field to 207 abstracts/texts for review, and ultimately found 9 relevant research studies for a final analysis. In these 9 studies, researchers in China, Colombia, Guatemala, Haiti, India, Mexico, Peru, and Vietnam used various intervention components, then followed clinical (such as anthropometrics and dietary intake), economic (such as morbidity and illness incidence), and process (such as retention and adherence) (Table 2). We examined strengths and weaknesses of each research program. While most studies included nutrition education components for caregivers (*n* = 7) (25, 30–35), no study included routine and frequent screening for nutrition risk in pediatric populations, only 4 included frequent follow-ups (25, 26, 32, 33), only 3 studies measured breast feeding frequency (25, 30, 35), and only 4 used supplemental nutrition with macronutrients + multiple micronutrients when indicated (25, 33, 35, 36). Only 4 studies reported economic outcomes (25, 26, 33, 36), though most (*n* = 7) reported process outcomes along with clinical outcomes (25, 26, 30, 32, 33, 35, 36).

**Table 2:** Recent community-based studies on nutritional interventions to improve pediatric stunting.

	Study design	Intervention components	Key results and conclusions
Castro Prieto et al. (30), Colombia	Malnutrition focus: stunting, wasting Duration: 10 months Child age: <10 months Inclusion criteria: at-risk-for stunting (≥ − 2 to <−1 HAZ) and/or chronic malnutrition (< −2 HAZ) *N* = 1,126 Type: single-arm public health intervention Setting: Community Center Intervention: *see next column* Control: N/A Primary outcome: nutritional status, anthropometrics, dietary intake Outcome types: clinical, process	Nutrient intake intervention: food vouchers for child and mother Education: nutrition (exclusive breastfeeding for children < 6 months, appropriate complimentary feeding for children > 6 months), parenting, child development, health care/child milestones, local resources Education mode: active (caregiver group sessions) Other resources provided: community empowerment Follow-up: non-frequent (baseline, 10 months)	Clinical 4.52% (*n* = 31) progressed from stunting to appropriate HAZ from baseline 21.57% (*n* = 146) progressed from at-risk for stunting to appropriate HAZ from baseline 37.80% of children < 6 months were exclusively breastfed, 73.70% continued breastfeeding past 6 months Process Overall retention: 60.9% (686/1126) Health care consultations adherence: 35.3% Food voucher adherence: 93.51%
Hall et al. (31), Vietnam	Malnutrition focus: stunting, wasting Duration: 18 months Child age: 6 years Child age: <10 months Inclusion criteria: children attending schools in regions with high prevalence of undernutrition *N* = 1,080 Type: controlled open-label parallel group Setting: school Intervention: 7 schools given fortified milk and biscuits, deworming Control: 14 schools with no feeding, deworming Primary outcome: anthropometrics Outcome types: clinical	Nutrient intake intervention: fortified food Education: health, nutrition, and hygiene Education mode: passive (flyers, posters) Other resources provided: deworming medication Follow-up: non-frequent (baseline, 18 months)	Clinical Children with less severe stunting at baseline gained slightly more height than children with more severe stunting (*p* = 0.011) When controlling for school, improved height in the intervention was not statistically significant (coefficient = −0.231, *t* = −0.79, *p* = 0.44) Control group also showed improvement in HAZ (*p* < 0.001) When height was the dependent variable in a regression model (*r* = 0.184, *r*^2^ = 0.034, adjusted *r*^2^ = 0.030, *F* = 9.367, *p* < 0.001), sex (*p* = 0.001) and initial HAZ score (*p* = 0.011) had a significant effect on height growth
Iannotti et al. (25), Haiti	Malnutrition focus: stunting, wasting Duration: 12 months Child age: 6–11 months Inclusion criteria: good health, singleton birth, WLZ > −3, household not receiving any food aid, residence within health center radius *N* = 589 Type: RCT with parallel design Setting: outpatient Intervention: (1) 3-month; and (2) 6-month lipid-based nutrient supplement (LNS) + education + standard of care of well-baby services Control: no LNS + education + standard of care with well-baby services Primary outcome: anthropometrics Outcome types: clinical, economic	Nutrient intake Intervention: Macronutrient + multiple micronutrient supplementation Education: nutrition (breastfeeding, complementary nutrition), and hygiene Education mode: active (caregiver group sessions) Other resources provided: standard of care with well-baby services Follow-up: frequent (baseline, 1, 2, 3, 4, 5, 6, and 12 months)	Clinical 3- month and 6-month group, respectively, when adjusted for child age had increased LAZ at 5 (*p* = 0.03, *p* = 0.02) and 6 months (*p* = 0.03, *p* = 0.04) Breastfeeding frequency improved growth in the intervention groups but was shown to be a significant negative mediating factor (Sobel-Goodman medication test) and was removed from the GLS model Economic Morbidity prevalence did not differ by study group (*p* > 0.05) Process 71% completed the study 98.0% (95% CI: 97.0–100.0%) and 97.0% (95% CI: 92.2–98.8%) of mothers in the 3-mo, and 6-mo LNS groups, respectively, consumed all LNS Adherence to proper administration of the LNS in both intervention groups was 6.0% (95% CI: 2.6–9.4%)
Juarez et al. (32), Guatemala	Malnutrition focus: stunting, wasting Duration: 2 years Child age: <5 years Inclusion criteria: attendees of Maya Health Alliance programming N = 125 households Type: single arm quality improvement intervention Setting: community Intervention: *see next column* Control: N/A Primary outcome: stunting prevalence, anthropometrics Outcome types: clinical, process	Nutrient intake Intervention: micronutrient supplementation, household food rations Education: nutrition Education mode: active (individual/family counseling by CHW) Other resources provided: medical care, growth monitoring, deworming medication Follow-up: frequent (baseline, monthly up to 2 years)	Clinical Stunting prevalence among sample fell from 42.4 to 30.6% Mean LAZ/HAZ increased among sample from −1.77 to −1.47 Proportion of children with stunting declined 17% (95% CI: 3–31%, *p* = 0.02) Mean HAZ/LAZ improved by 0.06 (95% CI: 0.003–0.12, *p* = 0.04) Improvement in stunting via generalized estimating equations (16, 95% CI: 2–30%, *p* = 0.02) Process Proportion of children receiving recommended growth monitoring improved during intervention period (indicated via run chart) Proportion of children receiving micronutrient supplements improved during intervention period (indicated via run chart)
Khadilkar et al. (33), India	Malnutrition focus: stunting, wasting Duration: 3 months Child Age: 4–6 years Inclusion criteria: WAZ between −1 and −2, good health *N* = 216 Type: controlled open-label parallel group Setting: outpatient Intervention: 45 g ONS + nutrition education Control: nutrition education Primary outcome: WAZ, weight gain in g/kg/day Outcome types: clinical, economic, process	Nutrient intake Intervention: macronutrient + multiple micronutrient supplementation Education: nutrition Education mode: active (individual/family counseling by RDN) Other resources provided: weekly telephone reminders to adhere to nutrition education Follow-up: frequent (baseline, 15 days, 1 month, 3 months)	Clinical Between group difference in HAZ at 30 (*p* < 0.0001) and 90 ((*p* < 0.0001) days Improvement in dietary intake for intervention group including carbohydrate (*p* < 0.0001), protein (p < 0.0001), fat (*p* < 0.0001), and energy (*p* < 0.0001) Economic Intervention group had fewer illness episodes Process >80% adherence to ONS
López de Romaña et al. (26), Peru	Malnutrition focus: stunting, wasting Duration: 6 months Child age: 6–12 months Inclusion criteria: region experiencing high prevalence of pediatric iron-deficiency anemia, stunting, vitamin A deficiency, acute respiratory infections, and diarrheal diseases *N* = 313 Type: RCT Setting: household Interventions: (1) daily dose of iron (DI); (2) daily dose of multiple macronutrients (DMM); (3) weekly dose of multiple micronutrients Control: placebo Primary outcome: anthropometrics Outcome types: clinical, economic, process	Nutrient intake Intervention: micronutrient supplementation Education: N/A Education mode: N/A Other resources Provided: daily process evaluation visits Follow-up: frequent (monthly for 6 months)	Clinical No significant differences in height gain of children among groups during the trial period Mean HAZ dropped significantly in DI and WMM groups, though no significant differences in the amount of change in mean HAZ during the 6 months across treatment groups Anemia was best controlled by DMM and DI, and was significantly lower at 6 months than WMM and control groups Economic No significant differences in prevalence of diarrheal or respiratory infections among groups Decrease of prevalence of fever in control (*p* < 0.001) and WMM (*p* = 0.05) groups Process 12.5% dropout rate with no differences between treatment groups
Perdomo et al. (34), Mexico	Malnutrition focus: stunting, wasting Duration: 4 months Child age: <5 years Inclusion criteria: low BMI-Z, low HAZ, low WAZ *N* = 113 Type: single arm program evaluation Setting: community Intervention: *see next column* Control: N/A Primary outcome: anthropometrics Outcome types: clinical	Nutrient intake intervention: micronutrient + probiotic supplementation, food assistance programs Education: nutrition, hygiene, culture, food preparation Education mode: active (monthly individual/family counseling by HCP) Other resources Provided: deworming program, farm animals, farming equipment, community gardens Follow-up: non-frequent (monthly for those children at greater social risk; baseline, 4 months for others)	Clinical Very short stature prevalence improved 10.6% (CI: 6.1–17.9) to 6.2% (CI: 2.9–12.5) Short stature category remained unchanged at 30.1% (CI: 22.2–39.3) 56.1% of children with initial diagnosis of low stature or risk of low stature improved their HAZ score
Rosado et al. (36), Mexico	Malnutrition focus: stunting, wasting Duration: 6 months Child age: 12–24 months Inclusion criteria: communities participating in the oportunidades program *N* = 186 Type: randomized placebo-controlled longitudinal trial Setting: community Health Centers Intervention: (1) oportunidades food supplement (OFS); (2) whole powdered milk (PM) Control: placebo Primary outcome: anthropometrics, morbidity Outcome types: clinical, economic, process	Nutrient intake intervention: macronutrient + multiple micronutrient supplementation Education: N/A Education mode: N/A Other resources provided: daily process evaluation visits Follow-up: non-frequent (baseline, 3 months, 6 months)	Clinical Change in height was significant in all groups (*p* < 0.01) OFS final HAZ was significantly higher from baseline (*p* < 0.05) No differences in height evaluation (adjusted and unadjusted) between groups at 6 months Hemoglobin increased significantly in OFS and PM groups from baseline (*p* < 0.05), though not significant at 6 months Economic No significant differences of gastrointestinal disease or respiratory disease among groups Children had similar increases in their mental and motor development across groups Process 17% lost to follow-up (38/224) > 90% supplement adherence among all groups
Wang et al. (35), China	Malnutrition focus: stunting, wasting Duration: 18 months Child age: 6–23 months Inclusion criteria: residency in national poverty county selected by Millenium Development goals Achievement Fund in China *N* = 693 Type: nutrition intervention program and single-arm clinical trial Setting: community health centers Intervention: *see next column* Control: N/A Primary outcome: anthropometrics Outcome types: clinical, process	Nutrient intake intervention: macronutrient + multiple micronutrient supplementation Education: nutrition (exclusive breastfeeding for children < 6 months, appropriate complimentary feeding for children > 6 months) Education mode: passive (TV broadcast, brochures, ‘11 types of training materials and 58,806 pieces of advocacy materials were distributed among the target areas and families’) Other resources Provided: N/A Follow-up: non-frequent (baseline, 18 months)	Clinical Improvement in LAZ (*p* < 0.05) at follow-up from baseline Decrease in stunting (*p* < 0.05), anemia (*p* < 0.05), vitamin B_12_ deficiency (*p* < 0.05), and vitamin A insufficiency (*p* < 0.05) at follow-up Risk of stunting only associated with a non-diverse diet (OR = 1.48, 95% CI:1.06–2.07, *p* < 0.05) Hemoglobin (*p* < 0.05), retinol (*p* < 0.05), ferritin (*p* < 0.05), folic acid (*p* < 0.05), and vitamin B_12_ (*p* < 0.05) improved at 18 months Consuming > 6 sachets/week had less anemia (p < 0.05), vitamin A insufficiency (p > 0.05), and iron deficiency (*p* = 0.06) No difference in breastfeeding at follow-up from baseline (*p* > 0.05) Process 15.8% (130/823) lost to follow-up Larger proportion of children liked supplement and consumed more than younger children (*p* < 0.05)

BMI-Z, BMI z-score; CHW, Community Health Worker; CI, Confidence Interval; GLS, generalized least-squares; HAZ, height-for-age z-score; HCP, Healthcare Professional; LAZ, length-for-age z-score; N/A, Not Applicable; ONS, Oral Nutrition Supplement; OR, Odds Ratio; RCT, Randomized Control Trial; RDN, Registered Dietitian Nutritionist; WAZ, weight-for-age z-score; WHZ, weight-for-height z-score; WLZ, weight-for-length z-score; non-frequent follow-up (greater than monthly/more than 4 weeks) frequent follow-up (at least once per month); active education was defined as synchronous education; passive education was defined as asynchronous education.

### 3.1 Studies involving multiple components (nutrition, education, other resources) had a synergistic and greater impact on outcomes (*n* = 7)

We found that studies using health education of caregivers or those using supplements fortified with multiple macronutrients and multiple micronutrients resulted in significant increases in height-for-age scores (37). Studies that included a nutritional, educational, and other resource components and/or frequent follow-ups showed greater improvements in stunting and other outcomes (25, 30–35). Studies that included only a nutritional and educational component with non-frequent follow-up had mixed results regarding improvement in stunting and/or wasting (31, 35, 36). The exact synergistic recipe of intervention components leading to greater results was unclear.

### 3.2 Studies with frequent follow-up had better outcomes (*n* = 4)

Most studies with frequent follow-up showed a trend of improvement in stunting outcomes (*n* = 3/4) (25, 32, 33), though it is important to note that the one study without good stunting outcomes included a supplement with micronutrients only but not additional resources (26). When reported, studies with frequent follow-up (at least once a month) also had good nutrition supplementation adherence (>80%) and low attrition rates. Those studies with less frequent follow-up (greater than 1 month between follow-ups) (30, 31, 34–36), had mixed results in whether stunting improvement was observed, and were mixed in their approach on length of frequency in between follow-ups, type of supplement, education, and other resources.

### 3.3 Comprehensive studies with macronutrient + multiple micronutrient supplements yielded better clinical outcomes (*n* = 4)

Children consuming macronutrient + multiple micronutrient supplements as part of a comprehensive nutrition intervention programs showed significant improvements in stunting (25, 33, 35), whereas when consuming a macronutrient + multiple micronutrient supplement without frequent follow-up and no education, improvements in stunting were not observed (36). This trend was similar among interventions that utilized micronutrient supplements or receiving food vouchers as part of comprehensive nutrition intervention programs (30, 32, 34). When studies did not utilize macronutrient + multiple micronutrient supplements or active education, there were no improvements in stunting (26, 31).

## 4 Discussion

The results of this review revealed that there is no single feeding strategy for addressing growth stunting in young children nor is there a universal best-practice package of interventions. However, our synthesis showed that multi-component interventions, those combining nutrition education, macronutrient and micronutrient supplementation, and regular follow-up, consistently led to better improvements in stunting-related outcomes.

Our review included studies from middle- and low-income countries around the world; however, the final selection was limited to studies from Latin America and South Asia due to the inclusion criteria. This geographic distribution reflects a limitation of available evidence, not of global need, and suggests a need for expanded research in other high-burden regions, particularly Sub-Saharan Africa and Southeast Asia (Table 2).

Although the interventions studied varied in scope and intensity, some consistent trends were observed. Studies that included multiple components, particularly those combining nutrition education with fortified supplements and frequent follow-up, reported more favorable changes in height-for-age scores, adherence, and retention. Conversely, studies that lacked any one of these components often had mixed or less pronounced results. However, the precise configuration of components yielding the greatest benefit remains unclear. Importantly, none of the included studies employed routine nutritional risk screening, and only four assessed economic outcomes, highlighting significant implementation gaps that future research should address.

Although many interventions had broader nutrition goals, our synthesis focused specifically on stunting, in line with the global emphasis on addressing chronic malnutrition. Although well-known regional programs such as the DREAM initiative in Malawi (38) and the Rainbow Project in Zambia (39) address important aspects of undernutrition, they were excluded from our analysis as they did not meet the full inclusion criteria, specifically, the combination of nutrition education, supplementation, and stunting outcomes. These efforts are nonetheless critical and offer complementary insights into ongoing nutrition and early childhood development interventions in Sub-Saharan Africa.

Although the UNICEF action framework (24) for complementary feeding encourages action by private institutions, the lack of knowledge about how to implement a multi-faceted intervention may impede those actions. Another recent review echoes this: creating and sustaining multi-sector partnerships to combat pediatric malnutrition has its challenges, and mirrors our review’s findings for the need for comprehensive, multi-faceted nutritional care programs (40).

**Figure 1:**
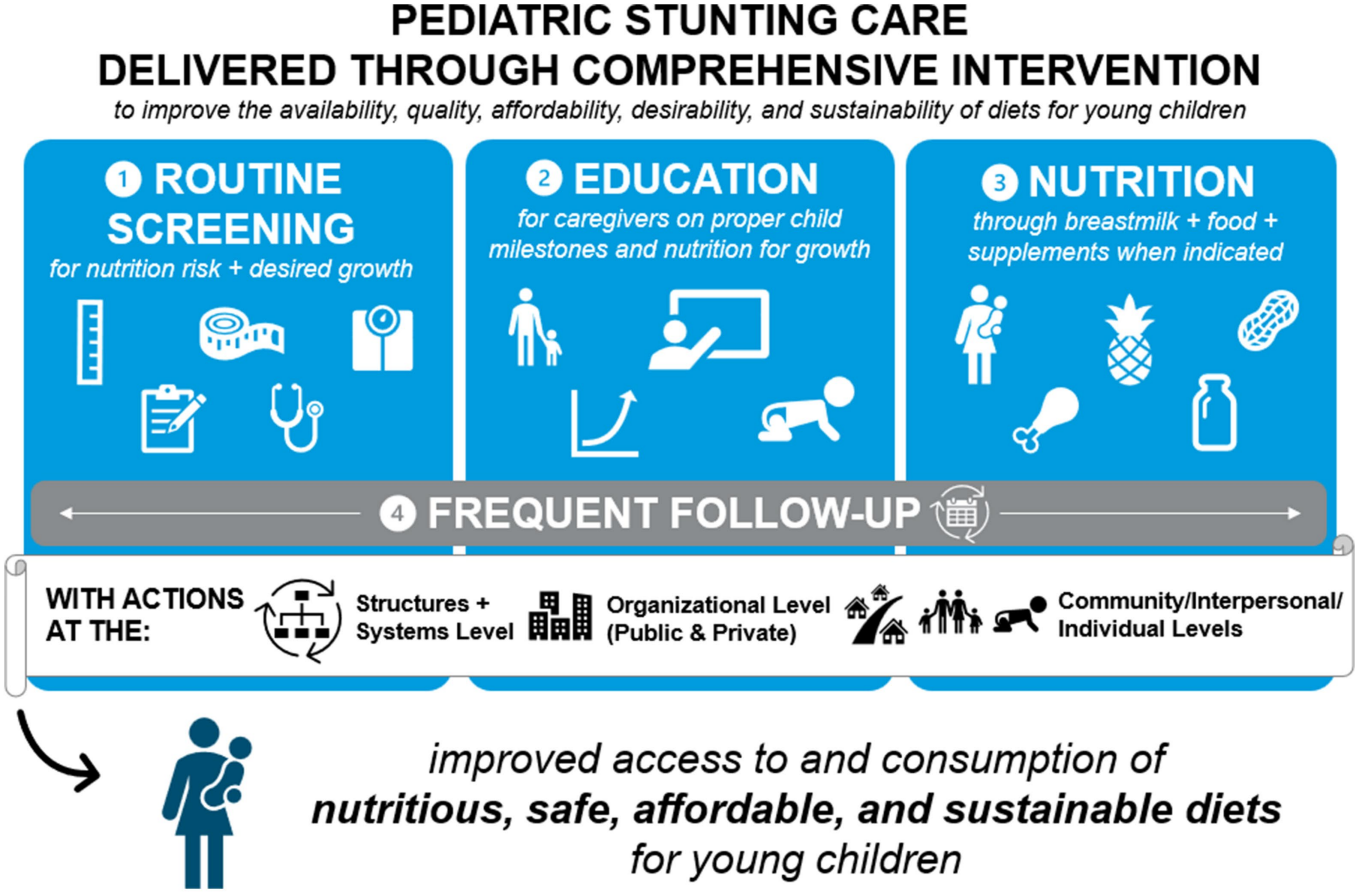
Pediatric stunting care delivered through comprehensive intervention.

With the development and evolution of sophisticated frameworks by WHO and UNICEF (Figure 1) (19, 21), as well as clearly enunciated principles, (21, 23) there is a clear call for multi-component approaches to impact the public health challenge of growth impairment due to nutritional insufficiency, as highlighted by our review. This call is echoed by a recent literature review highlighting how sustained, government-led, multi-sectoral strategies in Nepal, Bangladesh, and Vietnam have led to significant reductions in stunting prevalence over the past two decades (41). Our review supports this call for action, however, the exact recipe for synergistic intervention components with the most impact is unclear and future research should explore these components. Understanding these meaningful contributions by partners across different sectors are imperative to reduce stunting while improving the development of improved food environments, including their environmental sustainability, “to [create] sustainable, profitable models that explicitly include benefits to society and the environment” (23).

Guided by the frameworks of the United Nations, the World Health Organization, and the World Bank, we encourage stakeholders across multiple sectors to work together and implement nutrition-focused solutions at global, regional, and local levels. Such multi-component strategies and multi-level approaches are expected to lead to lasting and meaningful changes for care of children who are at risk of undernutrition and growth impairment (42, 43).

### 4.1 Multi-component and multi-level strategies for addressing growth stunting

Based on our review of strategies used by researchers across different care sites and world locations, we identified four key approaches to address growth stunting in young children, complemented by recommendations of the WHO and UNICEF. However, real-world implementation of these strategies faces substantial operational, cultural, and policy-level barriers, which must be addressed to optimize program impact.

#### 4.1.1 Regular nutritional screening

We underscore the importance of incorporating nutritional screening into regular community and clinical practices, especially in under-resourced communities where pediatric malnutrition/stunting is prevalent. Various screening tools are available to identify children at nutritional risk, defined as those due to their dietary characteristics, increased demands in their energy expenditure, or changes in anthropometric dynamics, have a greater probability of malnutrition, so selection can be tailored to meet needs by site and world region. Despite this, routine screening is rarely implemented though guidance from the WHO and UNICEF highlights the importance of screening for early identification. Logistical challenges, such as inadequate access to healthcare facilities and lack of calibrated measurement tools undermine screening efficacy (44). Additionally, workforce-related constraints, including limited personnel and insufficient training, further hinder consistent assessment and referral (45). These systemic limitations were also evident in our review, where none of the included studies integrated routine nutritional screening into the intervention protocol, highlighting a key gap that must be addressed to enable early identification and timely action.

#### 4.1.2 Community-engaged research

Engagement of local communities, particularly through partnerships with champions and leaders, plays a crucial role in improving intervention design, delivery and sustainability. We continue to emphasize the benefits of culturally competent care strategies that are developed in collaboration with the communities they aim to serve (43). Community involvement enhances program relevance and acceptability, ensures cultural alignment, and facilitates dissemination of findings in both local and scientific domains (46). However, gaining community buy-in remains a challenge, particularly when interventions are not perceived as culturally relevant. Qualitative research from rural Ethiopia revealed that low parental education, lack of nutrition knowledge, cultural norms, and gender inequalities were among the major barriers to appropriate child feeding, underscoring how such sociocultural factors can limit program participation and effectiveness (47). Additionally, in siloed operational environments, limited cross-sector collaboration and lack of community-based coordination can diminish the potential impact of such programs (48). To overcome these issues, it is critical to embed trust-building, cultural tailoring, and intersectoral alignment into community nutrition programs from the outset.

#### 4.1.3 Nutritional care with nutrition education + nutritional supplements

A key finding of our review was that interventions combining caregiver education with macro- and micronutrient supplementation were more effective in improving height-for-age z-scores than interventions using either strategy alone (5, 49). This combination was associated with improvements in both clinical outcomes and program retention.

However, real-world implementation of such combined approaches is frequently limited by resource constraints. Financial and supply-chain bottlenecks, including inconsistent delivery of supplements, inadequate infrastructure, and lack of regulatory incentives, undermine access and adherence (50, 51). Shortages of skilled personnel and limited parental knowledge also affect uptake (52, 53). Addressing these limitations through investments in workforce capacity, localized supply chain management, and culturally sensitive caregiver education can improve the reach and effectiveness of nutritional interventions.

#### 4.1.4 Follow-up with ongoing monitoring

Sustained follow-up is essential to assess intervention impact, reinforce caregiver behavior, and maintain adherence. Our review noted that programs with frequent follow-up and monitoring reported stronger gains in growth and retention (54). Follow-up activities should include anthropometric tracking, health and nutrition status checks, and evaluation of cost-effectiveness to ensure long-term feasibility (54).

Nonetheless, adherence to follow-up schedules remains a major challenge in many low-income settings. Program structure, such as inflexible scheduling, long travel distances, and limited home outreach contributes to poor attendance, with fewer than 14% of caregiver-child pairs in some settings completing the recommended number of visits (55). Socio-cultural factors, including stigma, body image concerns, and lack of health equity in care access, have been shown to contribute to non-compliance and early dropout in pediatric nutrition interventions (56). Additionally, systemic barriers, like weak policy frameworks, limited insurance coverage, and inadequate infrastructure, impede the establishment of robust follow-up mechanisms (57).

Addressing these challenges requires stronger institutional support, stakeholder engagement, and application of implementation science frameworks to scale follow-up protocols without compromising quality (58, 59). Tailoring monitoring systems to community needs and leveraging digital innovations, such as tools piloted in the INFANT and Nutrition Now programs, may further improve reach and sustainability (60, 61).

### 4.2 Limitations

One major limitation of this review is the small number of studies (*n* = 9) that met the inclusion criteria, which limits the generalizability of our findings. This restricted evidence base stems from the stringent inclusion criteria that required studies to include community-based, multi-component interventions specifically targeting stunting outcomes. While this approach enhanced relevance, it narrowed the pool of eligible studies. Future reviews with broader inclusion criteria or alternative designs may capture a wider spectrum of interventions and settings. This study is a narrative review informed by a systematic search, not a full systematic review. It did not employ a formal quality appraisal of included studies, and selection was purposive to capture illustrative examples of multi-component nutrition interventions addressing stunting.

## 5 Conclusion

Our review on nutritional care for children with undernutrition presenting as stunting identified studies involving a range of interventions at multiple socio-ecological levels. Based on these study findings, we propose that comprehensive approaches to care include routine screening for nutritional risk, nutrition education for caregivers, use of nutritional supplements with macro- and micro-nutrients when indicated, and frequent follow-up on growth and nutritional status.

Given the significant burdens that undernutrition and growth stunting impose on children and societies worldwide, future studies of such comprehensive nutritional care are needed to confirm the feasibility and effectiveness for improvement of clinical, health, and economic outcomes.
